# Supplementary material for: Early-life thymectomy leads to an increase of granzyme-producing γδ T cells in children with congenital heart disease
Source: Nat Commun. 2024 Nov 13;15:9841. doi: 10.1038/s41467-024-51673-3 (PMC11561289; doi:10.1038/s41467-024-51673-3)
Supplement: Supplementary file 1 — Supplementary Information [file 41467_2024_51673_MOESM1_ESM.pdf]

Early-life thymectomy leads to an increase of granzyme-producing γδ T cells in children with congenital heart disease

Alexa Cramer[1,‡], Tao Yang[1,‡], Lennart Riemann[1,2], Vicente Almeida[1], Christoph Kammeyer[3], Yusuf E Abu[1], Elisa Gluschke[3], Svea Kleiner[3], Ximena León-Lara[1], Anika Janssen[1], Alejandro Hofmann[4], Alexander Horke[5], Constantin von Kaisenberg[6], Reinhold Förster[1,7], Philipp Beerbaum[3], Martin Boehne[3,*], Sarina Ravens[1,7,*]

Affiliations

[1] Institute of Immunology, Hannover Medical School, Hannover, Germany

[2] Department of Pediatric Pneumology, Allergology and Neonatology, Hannover Medical School, Hannover, Germany

[3] Department of Pediatric Cardiology and Intensive Care Medicine, Hannover Medical School, Hannover, Germany

[4] Department of Pediatric Surgery, Hannover Medical School, Hannover, Germany.

[5] Department of Cardiothoracic, Transplantation and Vascular Surgery, Hannover Medical School, Hannover, Germany

[6] Department of Obstetrics, Gynecology and Reproductive Medicine, Hannover Medical School (MHH), Hannover, Germany

[7] Cluster of Excellence RESIST (EXC 2155), Hannover Medical School, 30625 Hannover, Germany

[‡] Equally contributing authors

* Corresponding authors

Email: ravens.sarina@mh-hannover.de; boehne.martin@mh-hannover.de

Supplementary Figure 1

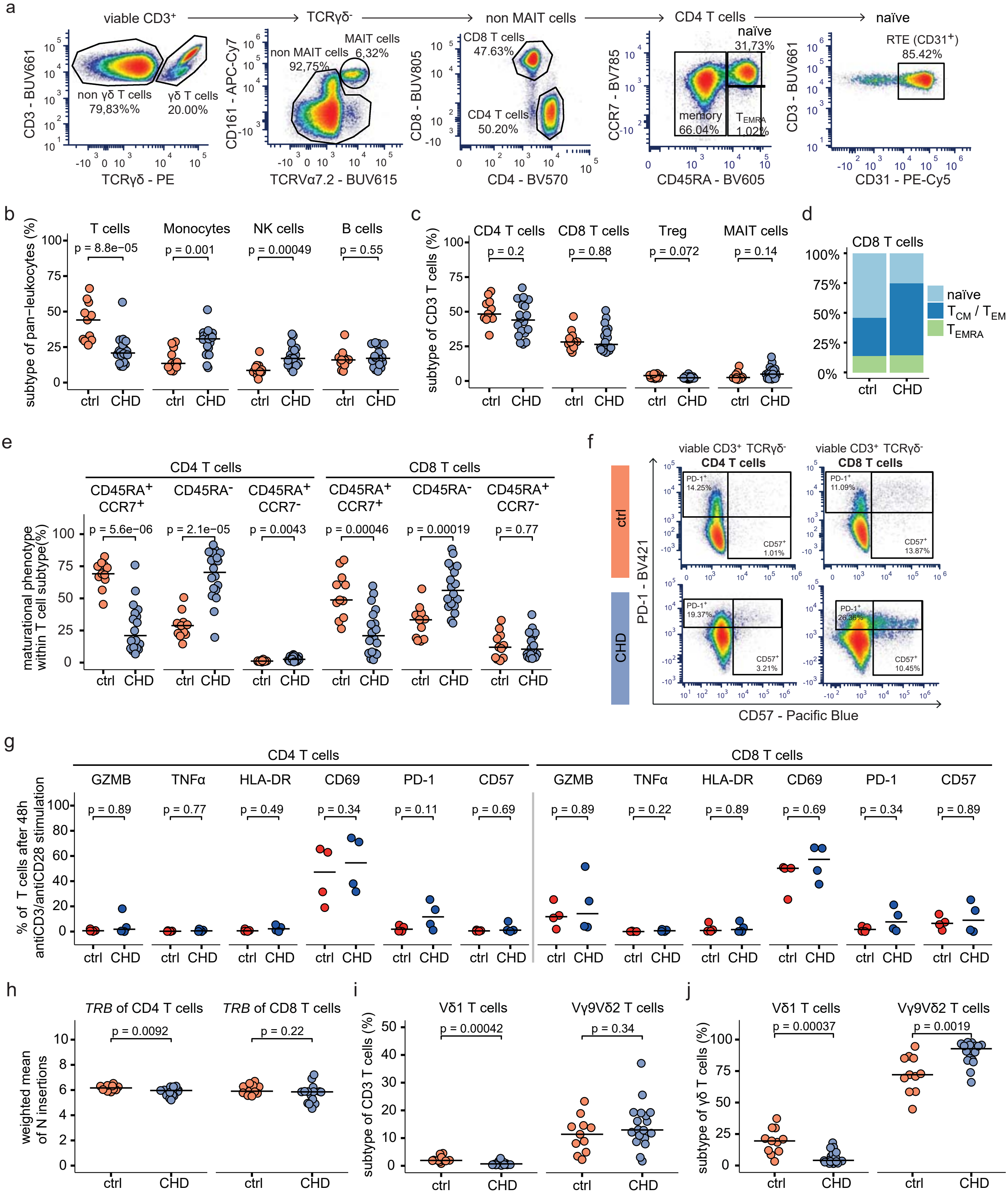

Supplementary Fig. 1, related to Fig. 1. αβ and γδ T cells in thymectomized children with CHD.

a) Gating strategy on one representative control sample to define T cell subsets, naïve and effector CD4 T cells, and recent thymic emigrants (RTE). **b**) Dot plots showing the percentage of $CD3^{+}$ T cells, $CD3^{neg}CD19^{neg}$ $CD14^{+}$ or/and $CD16^{+}$ monocytes, $CD3^{neg}CD19^{neg}CD56^{+}CD16^{+}$ NK cells and $CD19^{+}$ B cells within Zombie NIR^{neg} pan-leukocytes (defined by size within SSC-A and FSC-A). **c**) Percentage of respective T cell subsets ($CD4^{+}$, $CD8^{+}$, $CD4^{+}CD25^{+}CD127^{neg}$ (Tregs), or $CD161^{+}TCRVa7.2^{+}$ (MAIT cells)) within CD3 T cells. **d**) Stacked bar plot showing the median percentage of naïve ($CD45RA^{+}CCR7^{+}$), memory ($CD45RA^{-}$, either $CCR7^{+}$ (T_{CM}) or $CCR7^{-}$ (T_{EM})) or terminally differentiated effector memory phenotypes (T_{EMRA}; $CD45RA^{+}CCR7^{-}$) among CD8 T cells. **e**) Dot plots show the percentage of $CD45RA^{+}CCR7^{+}$, $CD45RA^{neg}$ or $CD45RA^{+}CCR7^{neg}$ cells among CD4 or CD8 T cells within ctrl and CHD children. **f**) Representative FACS plot of PD1 and CD57 expression on CD4 or CD8 T cells in one child with CHD and one control child. **g**) Frequency of cells expressing intracellular GZMB or TNFa, and surface HLA-DR, CD69, PD-1 or CD57 at 48 hours (h) post anti-CD3 and anti-CD28 within alive CD4 or alive CD8 T cells. **h**) N insertion counts in *TRB* sequences of either isolated CD4 or CD8 T cells, calculated as weighted mean, weighted by the total number of clones per sample. **i**) Frequency of $γδTCR^{+}$ Vδ1 T cells or Vγ9Vδ2 T cells within $CD3^{+}$ T cells as determined by FACS analysis. **j**) Frequency of Vδ1 T cells or Vγ9Vδ2 T cells within CD3+ γδ T cells. Statistical analyses were performed using the two-sided Wilcoxon-Mann-Whitney *U* test. Horizontal bars indicate median values. Each dot represents one donor. Source data are provided as a Source Data file.

Supplementary Figure 2

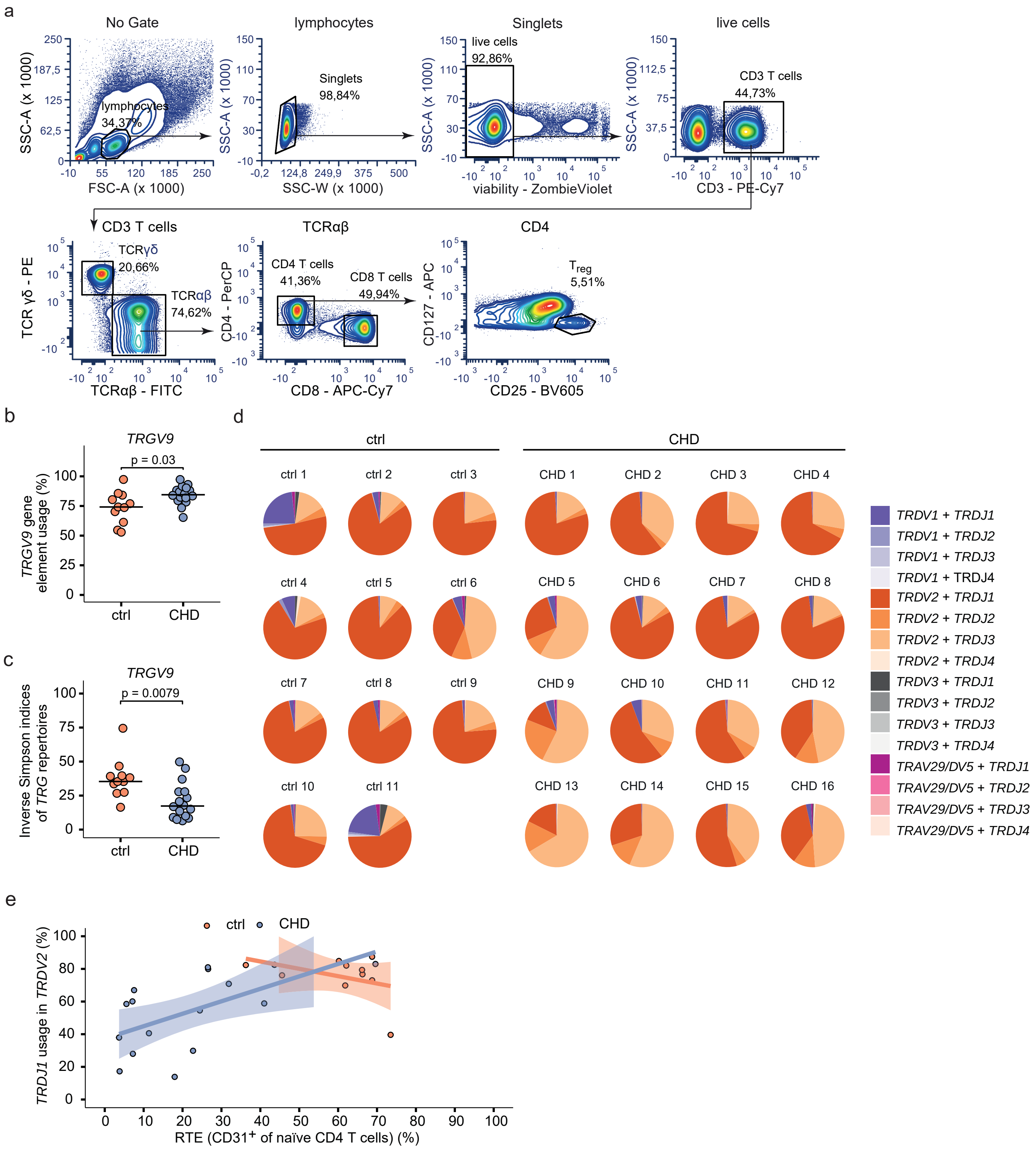

Supplementary Fig. 2, related to Fig. 1 and Fig. 2. TCR-seq analysis of γδ T cells in children with CHD.

TRG and *TRD* repertoire analysis of isolated blood γδ T cells from children with CHD (CHD) and controls (ctrl). **a**) Representative gating strategy to isolate γδ T cells and αβ T cells by FACS-sorting for TCR-seq. **b**) Percentual *TRGV9* gene usage within all *TRG* clones in the respective group. **c**) Inverse Simpson indices of *TRGV9*$^{+}$ sequences in the respective groups. **d**) Individual distribution of paired *TRDV* and *TRDJ* gene elements are depicted in pie charts for all control (ctrl) and all children with CHD (CHD). The size of pie slices represent the calculated frequency of respective gene pairs within the *TRD* repertoire. **e**) Percentage of RTE (CD31^{+} cells of naïve CD4+ T cells) plotted against the proportion of *TRDJ1*$^{+}$ within *TRDV2*$^{+}$ sequences for children with CHD (Spearman´s rho = 0.67, p-value = 0.0059) and controls (Spearman´s rho = -0.35, p-value = 0.3305). Statistical analyses were performed using the two-sided Wilcoxon-Mann-Whitney *U* test. Bars indicate median values; each dot represents for one donor. Source data are provided as a Source Data file.

Supplementary Figure 3

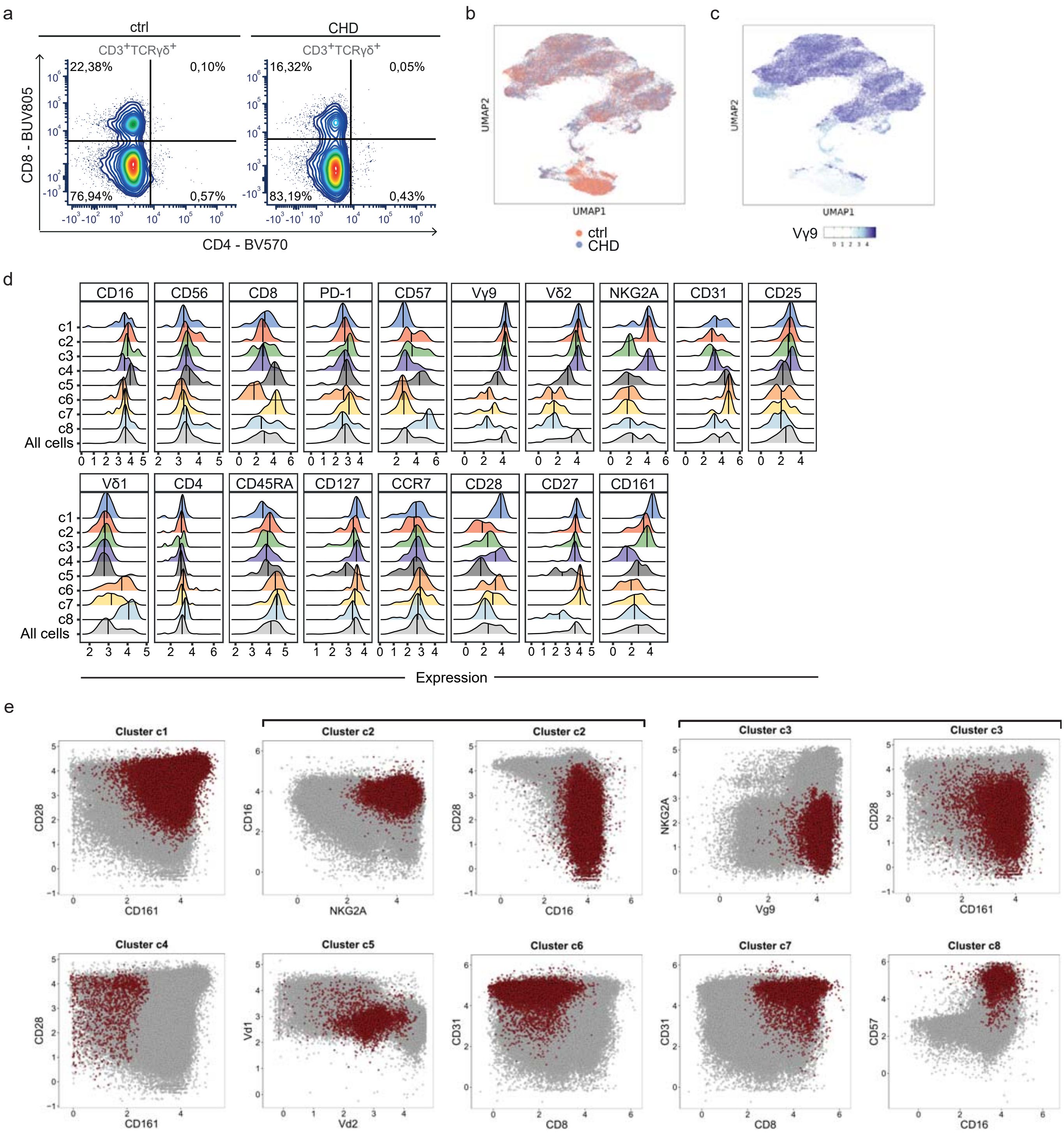

Supplementary Fig. 3, related to Fig. 3. Flow cytometric analyses of γδ T cells in children with CHD.

Flow cytometry data of pre-gated live $CD3^+TCR\gamma\delta^+$ cells from children with thymectomy (CHD, n = 18) and non-CHD controls (ctrl, n = 11). **a**) Representative manual gating of live CD3+TCRγδ+ cells for CD4 and CD8 surface marker expression. UMAPs of the obtained clusters are coloured by **b**) origin from either CHD or non-CHD control group (ctrl), and **c**) Vγ9 surface marker expression. **d**) Ridge plots showing the expression surface markers used for unsupervised clustering per cluster (c1-c8). Vertical lines indicate the median marker expression within each cluster. **e**) Scatterplots colored by marker expression of cells assigned to the obtained clusters c1-c8. Clusters are represented in one or two scatter plots, depending on the number of their most distinctive markers.

Supplementary Figure 4

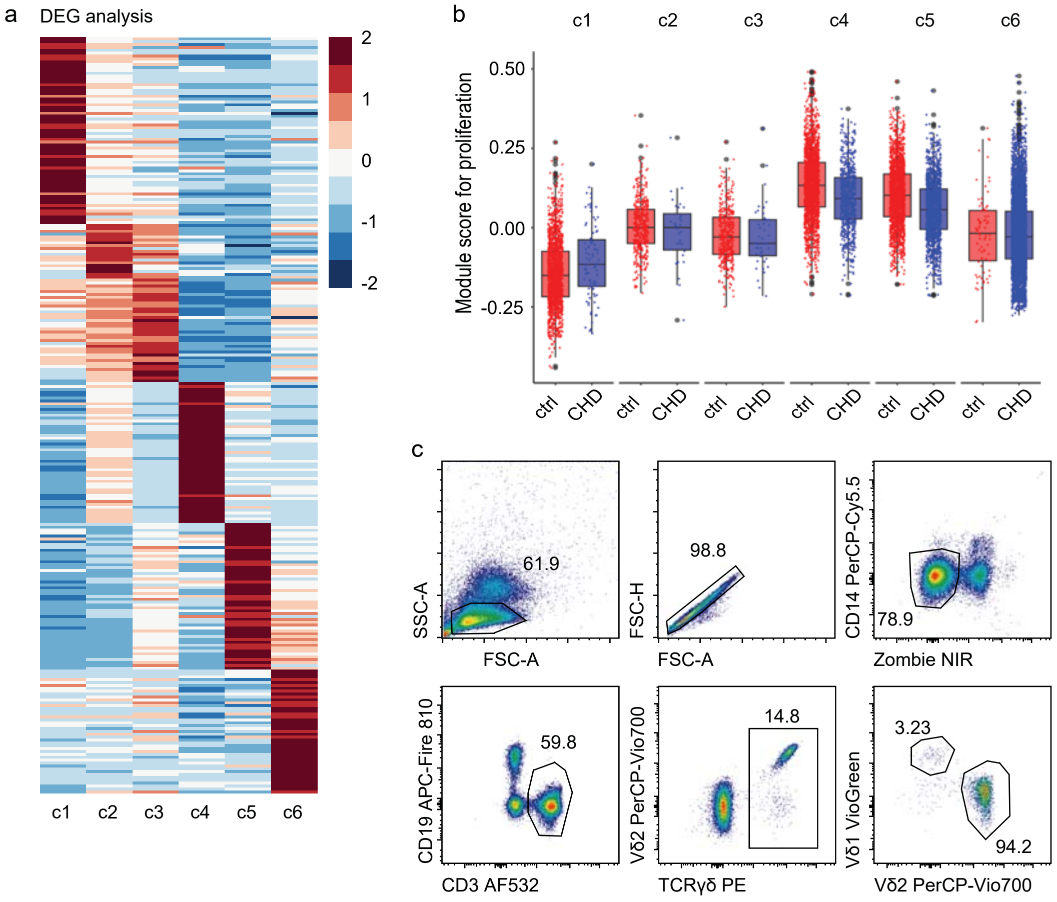

Supplementary Fig. 4, related to Fig. 4. scRNA sequencing of γδ T cells in children with CHD and age-matched controls. a) Heat map represents the top 50 DEGs for each identified cluster c1-c6. **b**) Box plots of the single-cell gene signature module score for proliferation per cluster and group, based on gene list: *CBLB*, *PTPN6*, *IRF1*, *HLA-DPB1*, *HLA-DPA1*, *HLA-DRB1, CCL5, TNFRSF1B, SH2D2A, FYN, BTN3A1, IL12RB1, SPN, CD81, MSN, ANXA1, SASH3, PYCARD, PTPN22, RASAL3, SELENOK, TNFSF14, ITCH, HLA-A, PSMB10, PTPRC, HLA-E, TNFRSF14, IL2RA, CD40LG, TNFRSF4, CR1, ANXA1, XCL1, SOS1, IL6ST, CD55, PRNP*, and *PPP3CA.* **c**) FACS-gating strategy to determine GZMA and GZMB expression on Vδ1 T cells. Source data are provided as a Source Data file.

Supplementary Figure 5

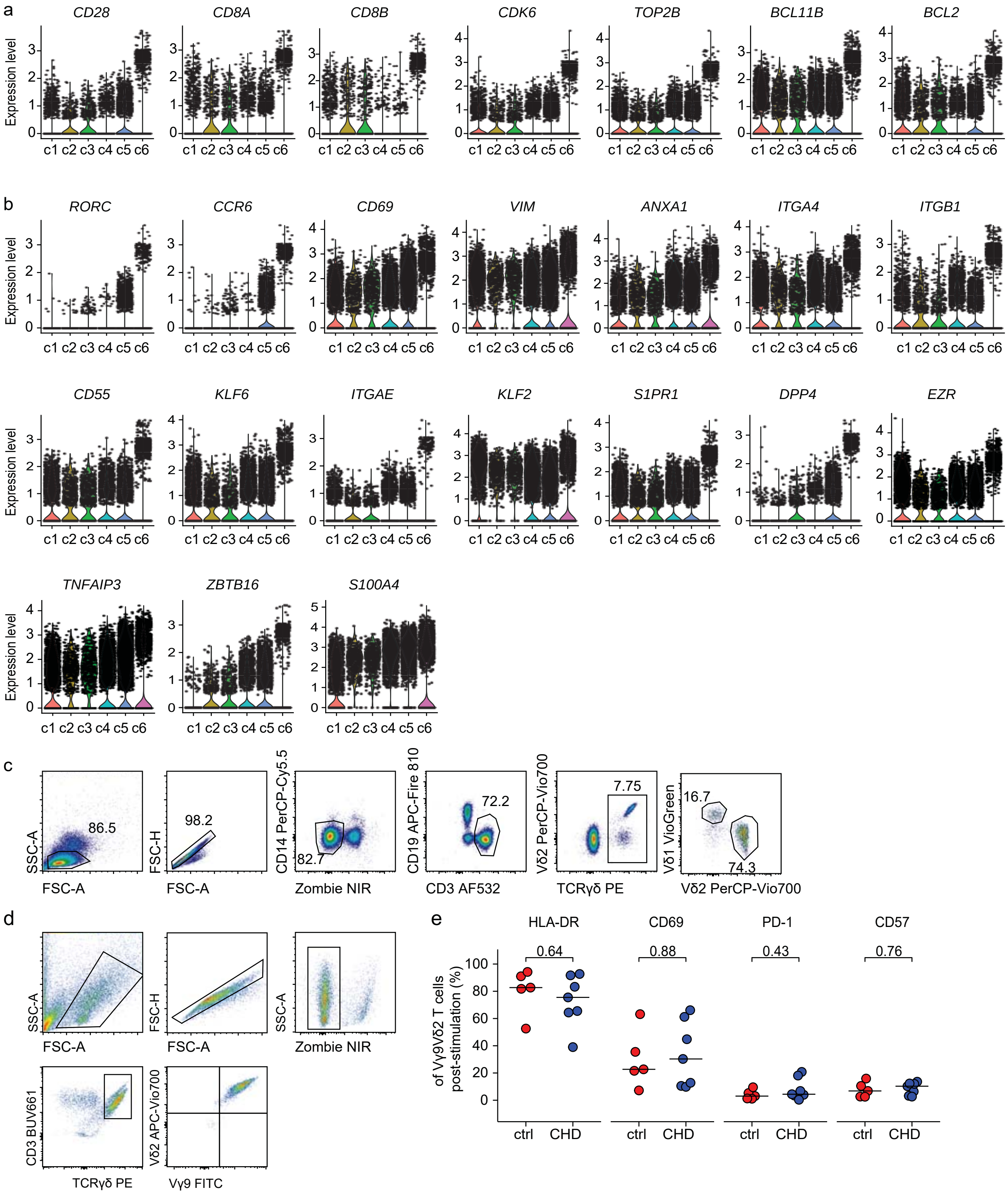

Supplementary Fig. 5, related to Fig. 5. Analyses of Vγ9Vδ2 T cells by sc-transcriptomics and in vitro stimulation in children with CHD.

(**a-b**) The gene expression of indicated genes within each cluster. **c**) Representative FACS gating strategy to determine GZMA, GZMB, CD28 and CD8A expression on Vδ2 T cells. **d**) Representative FACS gating strategy after 7 days in vitro HMBPP stimulation. **e**) Frequency of Vγ9Vδ2 T cells expressing HLA-DR, CD69, PD-1 and CD57 at day 7 post-stimulation. Source data are provided as a Source Data file.

Supplementary Figure 6

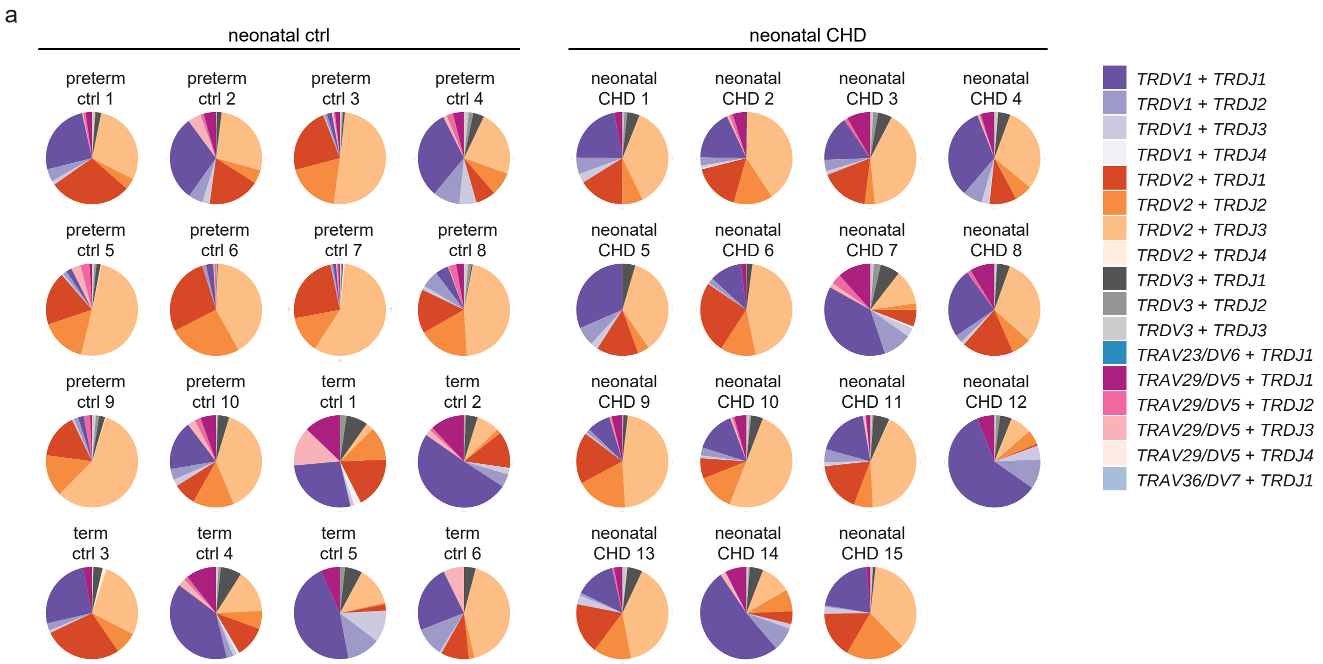

Supplementary Fig. 6, related to Fig. 6. TRD repertoire analyses in neonates prior surgery and age-matched controls.

TRD repertoire analysis from neonates with CHD prior cardiac surgery (nCHD, n = 15) and neonatal controls (ctrl, n = 16). **a**) Pie charts, color-coded by *TRDV-TRDJ* pairing within *TRD* repertoires, illustrating the percentual distribution of *TRDV-TRDJ* pairs per individual. For the ctrl group, the gestational age at birth is indicated as either term or preterm. Source data are provided as a Source Data file.

Supplementary Figure 7

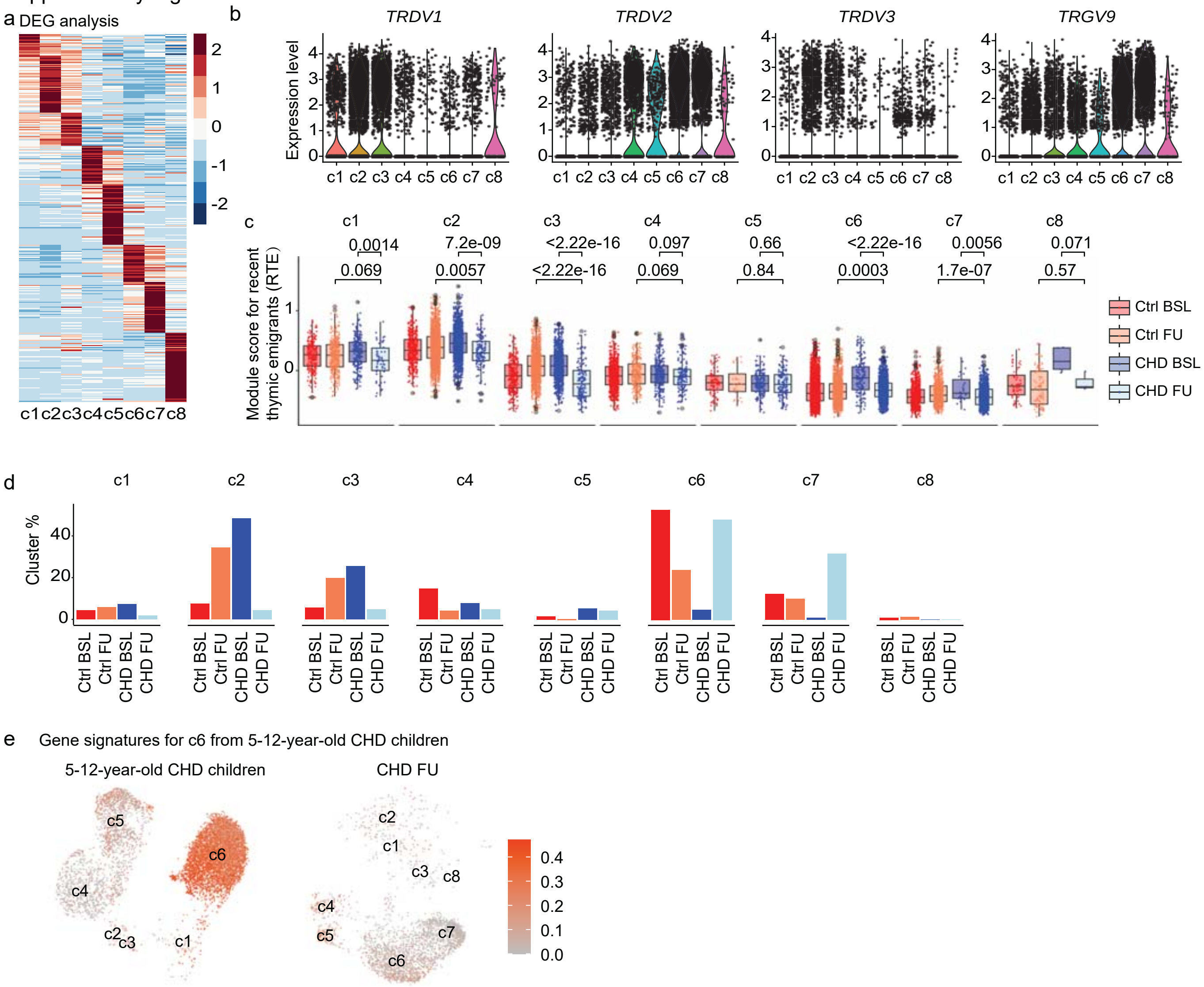

Supplementary Fig. 7, related to Fig. 7: Longitudinal single-cell transcriptomic analysis in infants with CHD pre- and post- cardiac surgery.

a) Heat map represents the top 50 DEGs for each identified cluster. **b**) The gene expression of *TRDV1-3* and *TRGV9* genes within each cluster. **c**) Box plots of the single-cell gene signature module score for recent thymic emigrants (RTE). The gene list is the same as Fig. 4e. Statistical significance was determined by unpaired two-sided Wilcoxon-Mann-Whitney *U* test. **d**) The bar plot reveals fractions of absolute cell numbers of each cluster from each group. **e**) Top 50 differentially expressed genes in $TRDV2^{+}$ cluster c6 of 5-12-year-old children with CHD were mapped to the transcriptional profiles of γδ T cells in the infants with CHD post-surgery (FU). Expression levels of gene signatures are displayed in the UMAP. Source data are provided as a Source Data file.

Supplementary Table S1, related to Table 1 and Table 2. Patient characteristics of early thymectomized children with CHD.

Age (yrs, mo)	Age at first surgery (days)	Congenital heart defect	Surgical and/or interventional procedures	Residual lesions	Conducted experiments
**5yr,8mo**	4d	HLHS MA/AA, ASD, duct-dependent systemic circulation	1. Stage 1 Norwood palliation, 2. PCPC, 3. Fontan	none	1, 3
**6yr,6mo**	2d	Heterotaxy syndrome, right isomerism, AVSD, TAPVR, DORV, PA, duct-dependent pulmonary circulation	1. mBTT shunt, 2. PCPC, 3. Fontan	none	1, 2, 3
**5yr,3mo**	7d	Shone's variant with hypoplastic LV, MS, hypoplastic aortic arch, CoA, multiple VSDs	1. Aortic arch reconstruction, partial VSD closure, 2. Tricuspid valve reconstruction, VSD + ASD enlargement, PAB, 3. Mitral valve reconstruction, VSD + ASD closure, debanding of PA	Mild MS	1, 2, 3
**5yr,6mo**	43d	Complex TGA/VSD/LVOTO, coronary artery anomaly	1. Ballon atrioseptostomy, 2. PDA stenting, 3. ASO, VSD + ASD closure	none	1, 2, 3
**5yr,4mo**	14d	Complex TGA/VSD/LVOTO	1. Ballon atrioseptostomy 2. mBTT shunt 3. Rastelli procedure, RV-PA conduit, ASD closure	Mild conduit stenosis	1, 2, 3, 4
**6yr,2mo**	28d	Shone's variant with hypoplastic LV, AS, hypoplastic aortic arch, CoA, VSD, ASD	1. Aortic arch reconstruction, aortic valve repair, ASD + VSD closure	none	1, 2, 3
**6yr,4mo**	16d	DORV, PA, duct-dependent pulmonary circulation	1. mBTT-Shunt, 2. RV-PA conduit, VSD closure	Moderate to severe PS, PH	1, 2, 3
**7yr,10mo**	5d	Simple d-TGA	1. Ballon atrioseptostomy 2. ASO	Severe PH	1, 2, 3
**10yr,3mo**	1d	Infracardiac TAPVR	1. TAPVR repair	None	1, 2, 3
**10yr,7mo**	10d	PA IVS, coronary sinusoids, ASD II, duct-dependent pulmonary circulation	1. mBTT-Shunt 2. PCPC 3. Fontan 4. Subaortic membrane resection	none	1, 2, 3
**10yr,2mo**	11d	Shone's variant with hypoplastic LV, MS, AS, hypoplastic aortic arch, CoA, VSD	1. Aortic arch reconstruction, repair CoA, VSD closure 2. Subaortic membrane resection, aortic valve commissurotomy 3. Subaortic membrane resection	LVOTO, moderate AS, moderate AR	1, 2, 3
**10yr,9mo**	13d	DORV with subpulmonary VSD, malposition of great arteries, hypoplastic aortic arch, duct-dependent systemic circulation	1. Ballon atrioseptostomy 2. ASO, aortic arch reconstruction, VSD + ASD closure	Moderate AR, moderate MR	1, 2, 3
**11yr,0mo**	3d	Simple d-TGA, ASD II	ASO	Mild PS	1

**11yr,7mo**	8d	Simple d-TGA, single sinus coronary artery with intramural course	1. Ballon atrioseptostomy 2. Mustard procedure	Mildly abnormal RV EF	1, 2, 3
**11yr,10mo**	40d	Taussig-Bing anomaly, RPA stenosis	1. ASO, VSD + ASD closure 2. RPA stenting	Mild RPA stenosis	1, 2, 3
**12yr,0mo**	7d	Simple d-TGA, ASD II	1. ASO	Moderate PS, mild AR	1, 2, 3
**12yr,9mo**	7d	Simple d-TGA, coronary anomaly	1. Ballon atrioseptostomy 2. ASO	Mild PR, Mild AR	1
**12yr,11mo**	9d	Simple d-TGA	1. Ballon atrioseptostomy, PDA stenting 2. ASO	Mild PS	1, 2, 3, 4
**7y, 7m**	7d	Simple d-TGA, ASD II, coronary artery anomaly	1. ASO	Mild RPA stenosis	4
**7y, 5m**	6d	Complex TGA/VSD	1. Balloon atrioseptostomy, 2. ASO, VSD closure	Mild PS	4

The study cohort consists of children aged 5 to 12 years with CHD who underwent partial or complete thymectomy within the first 6 weeks of life performed during palliative or corrective heart surgery. These children visited the pediatric cardiology outpatient clinic for a routine follow-up appointment. Aged-matched, generally healthy children planned for minor surgery served as controls (non-CHD ctrl).

Table shows baseline characteristics including age in years and month, height, weight at presentation, gender, congenital heart defect, age at (first) congenital heart surgery with concomitant thymectomy, surgical and/or interventional procedures in consecutive order, residual cardiac lesions and usage for different experimental settings (1 = Multicolor flow cytometry and FACS, 2 = *TRB* TCR-sequencing, *3* = *TRG* and *TRD* TCR-sequencing, 4 = single-cell RNA sequencing)

AA, aortic valve atresia; AR, aortic valve regurgitation; AS, aortic stenosis; ASD, atrial septal defect; ASO, arterial switch operation; mBTT, modified Blalock-Taussig-Thomas shunt; CoA, coarctation of aorta; DORV, double outlet right ventricle; d-TGA, dextro-transposition of great arteries; EF, ejection fraction; HLHS, hypoplastic left heart syndrome; LV, left ventricle; LVOTO, left ventricular outflow tract obstruction; MA, mitral atresia; MR, mitral regurgitation; MS, mitral stenosis; PA, pulmonary atresia; PAB, pulmonary artery banding; PA IVS, pulmonary atresia with intact ventricular septum; PAH, pulmonary arterial hypertension; PCPC, partial cavopulmonary connection; PDA, patent ductus arteriosus; PR, pulmonary valve regurgitation; PS, pulmonary stenosis; RPA, right pulmonary artery; RV, right ventricle; TAPVR, total anomalous pulmonary vein return; VSD, ventricular septal defect.

Supplementary Table 2, related to Table 3. Patient characteristics of neonates with critical CHD.

Age at surgery (days)	Congenital heart defect	Conducted experiments
**11d**	Neonatal critical AS	1
**14d**	Simple d-TGA with coronary anomaly, ballon atrioseptostomy	1
**6d**	Simple d-TGA, ballon atrioseptostomy	1
**5d**	Simple d-TGA	1
**5d**	Simple d-TGA	1
**4d**	Obstructed supracardiac TAPVR	1
**8d**	Simple d-TGA, ballon atrioseptostomy	1, 2
**9d**	Simple d-TGA	1, 2
**13d**	TAC A1	1
**15d**	IAA Typ A, duct-dependent systemic circulation	1
**3d**	HLHS MA/AA, restrictive IAS, duct-dependent systemic circulation, ballon atrioseptostomy	1
**8d**	Simple d-TGA	1
**7d**	Anomalous origin of the right pulmonary artery from the ascending aorta (AORPA)	1
**9d**	Simple d-TGA, ballon atrioseptostomy	1
**10d**	Shone's variant with hypoplastic LV, MS, AS, CoA,VSD, duct-dependent systemic circulation	1

Neonates with critical CHD were included on the day of planned congenital heart surgery. Demographic characteristic including age in days, congenital heart defect and usage for different experimental settings (1 = TCR-sequencing, 2 = longitudinal single cell RNA sequencing prior to surgery and 6 months after surgery) are shown. AA; aortic valve atresia; AORPA, anomalous origin of the right pulmonary artery from the ascending aorta; AS, aortic stenosis; ASO, arterial switch operation; CoA, coarctation of the aorta; d-TGA, dextro-transposition of the great arteries; HLHS, hypoplastic left heart syndrome; IAA, interrupted aortic arch; MA, mitral atresia; TAC, truncus arteriosus communis; TAC, truncus arteriosus communis; TAPVR, total anomalous pulmonary vein return: VSD, ventricular septal defect.

Supplementary Table 3, related to Figure 3. FACS antibody panel for immune cell phenotyping of PBMCs in children with CHD and age-matched controls.

**Fluorochrome**	**Marker**	**Clone**	**Company**	**Dilution**
BUV661	CD3	UCHT1	BD Horizon	1:100
BV570	CD4	RPA-T4	BioLegend	1:100
BUV805	CD8α	SK1	BD Horizon	1:100
PE-Cy	CD19	HIB1	eBioScience	1:200
BB700	CD14	MφP	BD	1:100
PE	TCRγδ	REA591	Miltenyi	1:200
VioGreen	Vδ1	REA173	Miltenyi	1:200
Per-CP-Vio700	Vδ2	REA771	Miltenyi	1:200
FITC	Vγ9	REA470	Miltenyi	1:200
BUV496	CD16	3G8	BD	1:100
BUV563	CD56	NCAM16.2	BD	1:100
BUV615	TCRVa7.2	OF-5A12	BD OptiBuild	1:100
APC-Cy7	CD161	HP-3G10	BioLegend	1:25
BV605	CD45RA	HI100	BioLegend	1:200
BV785	CCR7	G043H7	BioLegend	1:50
PE Fire 700	CD25	M-A251	BioLegend	1:100
AF700	CD127	O323	BioLegend	1:400
PE-Cy5	CD31	WM59	BioLegend	1:100
Alexa Fluor F700	CD27	O323	BioLegend	1:400
Alexa Fluor 647	CD28	CD28.2	BioLegend	1:200
APC-Fire810	CD38	HIT2	BioLegend	1:100
BV421	PD-1	EH12.2H7	BioLegend	1:25
PB	CD57	HNK-1	BioLegend	1:100
PE-Vio615	NKG2A	REA110	Miltenyi	1:200
Zombie NIR	Viability	-	BioLegend	1:800

Supplementary Table 4, related to Suppl. Fig. 1 and Figure 5. FACS antibody panel for to study surface molecules on HMBPP- or anti-CD3/anti-CD28-stimulated PBMC.

Fluorochrome	Marker	Clone	Company	Dilution
CellTrace™ Blue	Proliferation Tracking	-	Invitrogen	-
BUV563	CD8α	RPA-T8	BD	1:100
BUV661	CD3	UCHT1	BD Horizon	1:100
BUV737	CD69	FN50	BD	1:100
BV421	PD-1	EH12.2H7	BioLegend	1:25
PB	CD57	HNK-1	BioLegend	1:100
BV570	HLA-DR	L243	BioLegend	1:100
BV750	CD4	SK3	BioLegend	1:100
FITC	Vγ9	REA470	Miltenyi	1:100
PE	TCRγδ	REA591	Miltenyi	1:200
Zombie NIR	Viability	-	BioLegend	1:1000
APC-Vio770	Vδ2	REA771	Miltenyi	1:200

Supplementary Table 5, related to Suppl. Fig. 1 and Figure 5. FACS antibody for intracellular analysis of effector molecules in HMBPP- and anti-CD3/anti-CD28-stimulated PBMC.

Fluorochrome	Marker	Clone	Company	Dilution
**PE-Cy7**	IFN-γ	B27	BioLegend	1:100
**APC**	GZMB	GB12	Invitrogen	1:50
**AF700**	TNF-α	MAb11	BioLegend	1:50

Supplementary Table 6, related to Figure 4, 5. FACS antibody panel for analysis of GZMA and GZMB expression.

Fluorochrome	Marker	Clone	Company	Dilution
BUV805	CD8α	SK1	BD	1:100
VioGreen	Vδ1	REA173	Miltenyi	1:50
BV750	CD4	SK3	BioLegend	1:200
FITC	CD8β	S21011A	BioLegend	1:50
AF532	CD3	SK7	eBioscience	1:25
PerCP-Vio700	Vδ2	REA771	Miltenyi	1:200
PE	TCRγδ	REA591	Miltenyi	1:200
PE-Cy5	CD28	CD28.2	BioLegend	1:100
APC	GZMA	CB9	BioLegend	1:50
AF647	GZMB	GB11	BioLegend	1:50
Zombie Nir	Viability	-	BioLegend	1:1000
APC-Fire810	CD19	HIB19	BioLegend	1:100
