# Supplementary material for: Early-life thymectomy leads to an increase of granzyme-producing γδ T cells in children with congenital heart disease
Source: Nat Commun. 2024 Nov 13;15:9841. doi: 10.1038/s41467-024-51673-3 (PMC11561289; doi:10.1038/s41467-024-51673-3)
Supplement: Supplementary file 3 — Reporting Summary [file 41467_2024_51673_MOESM3_ESM.pdf]

nature portfolio

Corresponding author(s): Sarina Ravens, Martin Boehne

Last updated by author(s): 204-07-26

Reporting Summary

Nature Portfolio wishes to improve the reproducibility of the work that we publish. This form provides structure for consistency and transparency in reporting. For further information on Nature Portfolio policies, see our Editorial Policies and the Editorial Policy Checklist.

Statistics

For all statistical analyses, confirm that the following items are present in the figure legend, table legend, main text, or Methods section.

n/a	Confirmed	
☐	☒	The exact sample size (*n*) for each experimental group/condition, given as a discrete number and unit of measurement
☐	☒	A statement on whether measurements were taken from distinct samples or whether the same sample was measured repeatedly
☐	☒	The statistical test(s) used AND whether they are one- or two-sided<br>*Only common tests should be described solely by name; describe more complex techniques in the Methods section.*
☐	☒	A description of all covariates tested
☐	☒	A description of any assumptions or corrections, such as tests of normality and adjustment for multiple comparisons
☐	☒	A full description of the statistical parameters including central tendency (e.g. means) or other basic estimates (e.g. regression coefficient) AND variation (e.g. standard deviation) or associated estimates of uncertainty (e.g. confidence intervals)
☐	☒	For null hypothesis testing, the test statistic (e.g. *F*, *t*, *r*) with confidence intervals, effect sizes, degrees of freedom and *P* value noted<br>*Give P values as exact values whenever suitable.*
☒	☐	For Bayesian analysis, information on the choice of priors and Markov chain Monte Carlo settings
☒	☐	For hierarchical and complex designs, identification of the appropriate level for tests and full reporting of outcomes
☐	☒	Estimates of effect sizes (e.g. Cohen's *d*, Pearson's *r*), indicating how they were calculated

Our web collection on statistics for biologists contains articles on many of the points above.

Software and code

Policy information about availability of computer code

Data collection	No software was used.
Data analysis	For scRNA-seq data analysis, Cell Ranger 7.0 was used to generate the gene expression matrix. The subsequent analysis was performed using Seurat v5.0.17, Harmony, and clusterProfiler in R 4.3.2. For high-dimensional flow cytometry data analysis, Peak-based selection of high quality cytometry data (PeacoQC, 1.14.0) algorithm and Flow Self-Organizing Maps (FlowSOM, 2.12.0) algorithm were used. For TCRseq analysis, MiXCR 4.3.2 and Immunarch 1.0.0 were used.

For manuscripts utilizing custom algorithms or software that are central to the research but not yet described in published literature, software must be made available to editors and reviewers. We strongly encourage code deposition in a community repository (e.g. GitHub). See the Nature Portfolio guidelines for submitting code & software for further information.

Data

Policy information about availability of data

All manuscripts must include a data availability statement. This statement should provide the following information, where applicable:

- Accession codes, unique identifiers, or web links for publicly available datasets
- A description of any restrictions on data availability
- For clinical datasets or third party data, please ensure that the statement adheres to our policy

Raw FASTQ files of the TCR-seq data has been deposited at the NCBI SRA under the BioProject: PRJNA1040021. scRNAseq data are accessible through this link: https://zenodo.org/uploads/11243058.

Research involving human participants, their data, or biological material

Policy information about studies with human participants or human data. See also policy information about sex, gender (identity/presentation), and sexual orientation and race, ethnicity and racism.

Reporting on sex and gender	Gender is N.A. Sex is summarized in the corresponding tables 1-3.
Reporting on race, ethnicity, or other socially relevant groupings	N.A.
Population characteristics	Our study focused on investigating immunological changes in children who had undergone cardiac surgery of CHD with concomitant thymectomy and enrolled 5–12-year-old children after surgery (Table 1) and neonates before surgery (Table 3). The children and neonates were diagnosed with a wide spectrum of critical and/or complex CHDs, such as simplex d-TGA (dextro-transposition of great arteries), complex TGA, TAPVR (total anomalous pulmonary vein return), and Shone´s variant (Supplementary Table S1, Supplementary Table S2), in some cases requiring multiple surgical and/or interventional procedures.
Recruitment	For the post-surgical study population children with CHD (aged 5–12 years), who visited the pediatric cardiology outpatient clinic for a routine follow up appointment, were eligible for the study. These study participants had underwent partial or complete thymectomy within the first 6 weeks of life performed during a palliative or corrective heart surgery (n = 18, Supplementary Table S1). Age-matched, generally healthy children who had a blood sample taken before planned minor surgery at the Department of Pediatric Surgery, Hannover Medical School, Hannover, Germany, were included in the study as non-CHD controls.
Ethics oversight	Hannover Medical School (10198_BO_S_2022)

Note that full information on the approval of the study protocol must also be provided in the manuscript.

Field-specific reporting

Please select the one below that is the best fit for your research. If you are not sure, read the appropriate sections before making your selection.

☒ Life sciences ☐ Behavioural & social sciences ☐ Ecological, evolutionary & environmental sciences

For a reference copy of the document with all sections, see nature.com/documents/nr-reporting-summary-flat.pdf

Life sciences study design

All studies must disclose on these points even when the disclosure is negative.

Sample size	The sample size was determined based on patients who visited the hospital.
Data exclusions	No data was excluded.
Replication	Replication was done by different methods, namely FACS phenotyping, in vitro stimulation, and single cell RNA sequencing.
Randomization	Patients were selected based on the disease.
Blinding	The initial data analysis of all FACS data was blinded.

Reporting for specific materials, systems and methods

We require information from authors about some types of materials, experimental systems and methods used in many studies. Here, indicate whether each material, system or method listed is relevant to your study. If you are not sure if a list item applies to your research, read the appropriate section before selecting a response.

Materials & experimental systems

n/a	Involved in the study	
☐	☒	Antibodies
☒	☐	Eukaryotic cell lines
☒	☐	Palaeontology and archaeology
☒	☐	Animals and other organisms
☒	☐	Clinical data
☒	☐	Dual use research of concern
☒	☐	Plants

Methods

n/a	Involved in the study	
☒	☐	ChIP-seq
☐	☒	Flow cytometry
☒	☐	MRI-based neuroimaging

Antibodies

Antibodies used

CD3 BUV661, UCHT1, BD, Cat 612964, Lot 0311377;
CD4 BV570, RPA-T4, BioLegend, Cat 300534, Lot B346965;
CD8α BUV805, SK1, BD, Cat 612889, Lot 2140241;
CD19 PE-Cy, HIB1, eBioScience, Cat 25-0199-42, Lot 2527485;
CD14 BB700, MϕP, BD, Cat 566465, Lot 1055172;
TCRγδ PE, REA591, Miltenyi, Cat 130-113-512, Lot 5240102466;
Vδ1 VioGreen, REA173, Miltenyi, Cat 130-120-442, Lot 5221105125;
Vδ2 Per-CP-Vio700, REA771, Miltenyi, Cat 130-111-014, Lot 5230405826;
Vγ9 FITC, REA470, Miltenyi, Cat 130-125-204, Lot 5231106985;
CD16 BUV496, 3G8, BD, Cat 612944, Lot 2272394;
CD56 BUV563, NCAM16.2, BD, Cat 612928, Lot 0325598;
TCRVa7.2 BUV615, OF-5A12, BD, Cat 751162, Lot 2147028;
CD161 APC-Cy7, HP-3G10, BioLegend, Cat 339928, Lot B399062;
CD45RA BV605, HI100, BioLegend, Cat 304134, Lot B346157;
CCR7 BV785, G043H7, BioLegend, Cat 353230, Lot B340912;
CD25 PE Fire 700, M-A251, BioLegend, Cat 356146, Lot B376207;
CD31 PE-Cy5, WM59, BioLegend, Cat 303150, Lot B338135;
CD27 Alexa Fluor F700, O323, BioLegend, Cat 302814, Lot B316073;
CD28 Alexa Fluor 647, CD28.2, BioLegend, Cat 302954, Lot B350959;
CD38 APC-Fire810, HIT2, BioLegend, Cat 303550, Lot B359420;
PD-1 BV421, EH12.2H7, BioLegend, Cat 329920, Lot B398161;
CD57 PB, HNK-1, BioLegend, Cat 359608, Lot B331659;
NKG2A PE-Vio615, REA110, Miltenyi, Cat 130-120-035, Lot 5221010545;
CD69 BUV737, FN50, BD, Cat 612817, Lot 2122353;
HLA-DR BV570, L243, BioLegend, Cat 307638, Lot B363164;
Vδ2 APC-Vio770, REA771, Miltenyi, Cat 130-111-013, Lot 5231009636;
IFN-γ PE-Cy7, B27, BioLegend, Cat 506518, Lot B326674;
GZMB APC, GB12, Invitrogen, Cat MHGB05, Lot 2690614;
TNF-α AF700, MAb11, BioLegend, Cat 502928, Lot B347121;
CD8β FITC, S21011A, BioLegend, Cat 387303, Lot B400042;
CD3 AF532, SK7, eBioscience, Cat 58-0038-42, Lot 2338602;
GZMA APC, CB9, BioLegend, Cat 507220, Lot B316004;
GZMB AF647, GB11, BioLegend, Cat 515406, Lot B341642;
CD19 APC-Fire810, HIB19, BioLegend, Cat 302272, Lot B375854;
CD8α BUV563, RPA-T8, BD, Cat 612914, Lot 20322344;
CD4 BV750, SK3, BioLegend, Cat 344643, Lot B355409;
CD127 BV650, AO1905, BioLegend, Cat 351326, Lot B379697;
CD103 BV711, BER-ACT8, BD, Cat 563162, Lot 2033635;
CD28 PE-Cy5, CD28.2, BioLegend, Cat 302910, Lot B336927

Validation

N.A.

Plants

Seed stocks | *Report on the source of all seed stocks or other plant material used. If applicable, state the seed stock centre and catalogue number. If plant specimens were collected from the field, describe the collection location, date and sampling procedures.*

Novel plant genotypes | *Describe the methods by which all novel plant genotypes were produced. This includes those generated by transgenic approaches, gene editing, chemical/radiation-based mutagenesis and hybridization. For transgenic lines, describe the transformation method, the number of independent lines analyzed and the generation upon which experiments were performed. For gene-edited lines, describe the editor used, the endogenous sequence targeted for editing, the targeting guide RNA sequence (if applicable) and how the editor was applied.*

Authentication | *Describe any authentication procedures for each seed stock used or novel genotype generated. Describe any experiments used to assess the effect of a mutation and, where applicable, how potential secondary effects (e.g. second site T-DNA insertions, mosiacism, off-target gene editing) were examined.*

Flow Cytometry

Plots

Confirm that:

☒ The axis labels state the marker and fluorochrome used (e.g. CD4-FITC).

☒ The axis scales are clearly visible. Include numbers along axes only for bottom left plot of group (a 'group' is an analysis of identical markers).

☒ All plots are contour plots with outliers or pseudocolor plots.

☒ A numerical value for number of cells or percentage (with statistics) is provided.

Methodology

Sample preparation | PBMCs were thawed in 37°C water bath and stained with antibodies.

Instrument | Spectral flow cytometer Cytek Aurora using Spectroflo software (Cytek Biosciences)

Software | FCS Express, FlowJo, Peak-based selection of high quality cytometry data (PeacoQC) algorithm and Flow Self-Organizing Maps (FlowSOM) algorithm

Cell population abundance | Purity of FACS-sorted T cell subset was determined by short re-analysis by FACS. The purity was above 95% for all samples.

Gating strategy | All antibodies were tested by titration and if neccessary by FMO controls. Gating strategy was based on the positive and negative signals.

☒ Tick this box to confirm that a figure exemplifying the gating strategy is provided in the Supplementary Information.
